# Resveratrol alleviated muscle fiber type transformation in oxidative stressed bovine myotubes through Akt/FoxO signaling pathway

**DOI:** 10.5713/ab.250976

**Published:** 2026-05-07

**Authors:** Huixin Zuo, Jing Sun, Jiatong Cui, Xiaoyin Yang, Yunge Liu, Yimin Zhang, Lixian Zhu

**Affiliations:** 1 College of Food Science and Engineering, Shandong Agricultural University, Tai’an, China; 2 National R&D Center for Beef Processing Technology, Tai’an, China

**Keywords:** Akt/FoxO Signaling Pathway, Bovine Myotubes, Fiber Transformation, Oxidative Stress, Resveratrol

## Abstract

**Objective:**

This research sought to explore how oxidative stress influences the composition of muscle fiber types in bovine myotubes and to assess the potential protective effect of resveratrol (RES) via the modulation of the Akt/FoxO signaling pathway.

**Methods:**

Bovine myotubes were treated with hydrogen peroxide (H_2_O_2_) to induce oxidative stress, with or without co-treatment with RES. The analyses included metabolic and antioxidant enzyme activities, real-time quantitative polymerase chain reaction, and western blotting to evaluate fiber-type markers, mitochondrial biogenesis genes, and components of the Akt/FoxO pathway.

**Results:**

Oxidative stress led to a downregulation of slow-type myosin heavy chain (*MyHC*) and an upregulation of fast-type *MyHC*. The activity of key oxidative enzymes, including succinate dehydrogenase (SDH) and malate dehydrogenase, along with major antioxidant enzymes such as superoxide dismutase, glutathione peroxidase, and catalase. Concurrently, it enhanced lactate dehydrogenase activity and increased malondialdehyde levels, signifying increased lipid peroxidation. It also inhibited the Akt/FoxO pathway and downregulated genes associated with mitochondrial biogenesis (*NRF-1*, *TFAM*, *ATP6*). RES treatment significantly attenuated these changes. The activation of Akt by SC79 similarly alleviated oxidative stress-induced fiber-type transformation.

**Conclusion:**

Oxidative stress induces a transformation from slow to fast muscle fiber types in bovine myotubes, associated with the inhibition of the Akt/FoxO signaling pathway and a suppression in mitochondrial biogenesis. RES mitigates this shift in muscle fiber composition; its positive effects are linked to the reactivation of the Akt/FoxO signaling pathway and the enhancement of mitochondrial biogenesis.

## INTRODUCTION

Skeletal muscles account for approximately 40% of the body weight of mammals, which are comprised of different myofibers [1]. Skeletal muscle fibers are categorized into four major types—Type I, IIa, IIx, and IIb—based on the specific myosin heavy chain (MyHC) isoforms they express; these correspond functionally to slow oxidative, fast oxidative, fast intermediate, and fast glycolytic fiber phenotypes, respectively [2]. Type I fibers, characterized by high mitochondrial and myoglobin content, support aerobic energy production and are positively associated with meat tenderness and juiciness [3]. Sensory tenderness scores for the longissimus thoracis and rectus abdominis rose in parallel with the proportion of rapidly contracting, glycolytic IIb and IIx fibers [4]. Lu et al. [5] demonstrated in Chinese Qinchuan and Luxi cattle that an increased proportion of type I fibers improved beef tenderness. Consistent results were obtained by Hou et al. [6] across bovine, porcine, and avian studies, where a higher slow-twitch fiber percentage was associated with greater tenderness and lower drip loss. Postnatally, the overall muscle fiber count remains largely unchanged, yet fiber-type composition undergoes continuous remodeling. Myofiber types are generally transformed in the order MyHC I↔MyHC IIa↔MyHC IIx↔ MyHC IIb.

Oxidative stress represents a prevalent physiological stress response in animals and can be induced by various environmental challenges, including elevated temperatures, transport and pre-slaughter stress, and nutritional restriction etc [7]. Redox imbalance induces skeletal muscle wasting mainly by expediting proteolysis and suppressing anabolism. This occurs via oxidative modification of myofibrillar proteins and activation of signaling cascades that regulate muscle-protein turnover. The forkhead box O (FoxO) family comprises transcription factors that orchestrate broad transcriptional programs, modulate cellular metabolism, and integrate adaptive responses to environmental perturbations—such as growth factor withdrawal and oxidative stress [8]. FoxO1 and FoxO3 (also known as FoxO3a) are well-known members of the FoxO family and the most frequently concerned [9]. Over-expression of FoxO1 via plasmid transfection in mice C2C12 cells enhances fast-twitch and suppresses slow-twitch fibers, indicating that FoxO1 facilitates the slow-to-fast transition [10].

Russell and Schneider [ 11 ] further demonstrated that Akt-dependent phosphorylation of FoxO affected its downstream transcriptional activity. Notably, Dodd et al. [ 12 ] demonstrated experimentally that reactive oxygen species (ROS) directly trigger FoxO signaling activation in skeletal muscle under conditions of disuse-induced atrophy, thereby revealing a causal molecular connection between oxidative stress and FoxO-driven structural and functional adaptations in muscle tissue. Collectively, these findings establish the Akt/FoxO signaling axis as a critical determinant of myofiber-type plasticity. Recent comprehensive reviews have further elaborated on the intricate roles of redox signaling in skeletal muscle remodeling through the Akt/FoxO pathway [ 13,14]. Given the negative impact of oxidative stress on meat quality, effective interventions are urgently needed. Recent studies have shown that the natural polyphenol resveratrol (RES) can ameliorate oxidative damage and modulate myofiber-type conversion through its potent antioxidant properties, thereby offering a promising strategy for meat-quality improvement [ 14 ].

RES, chemically identified as 3,4′,5-trihydroxystilbene, is a naturally occurring polyphenol prevalent in berries, peanuts, and grape-derived products. It is usually extracted from roots, stems, leaves, flowers, fruits and seeds. Results showed that dietary supplementation with RES markedly elevated crude protein and myoglobin concentrations in the longissimus dorsi muscle of finishing pigs, while also boosting antioxidant enzyme activities and upregulating the mRNA expression of MyHC IIa [ 16 ]. As a result, this reduced the shear force, water loss, lipid peroxide content, and improved the quality of pork. Many studies indicated that RES activated the Akt/FoxO signaling pathway—via SIRT1/Akt/FoxO1 in high-glucose myogenic induction and via Akt-mediated phosphorylation of FoxO1 that repressed its transcriptional activity [11,17]. Our novel findings demonstrate that RES induces a shift in muscle fiber composition—both in bovine skeletal muscle and in cultured bovine myotubes—from fast-twitch toward slow-twitch phenotypes, primarily through activation of the AMPK/SIRT1/PGC-1α signaling axis [18]. Specifically, when the AMPK inhibitor Compound C was added, the muscle fiber type-transforming effect of RES was significantly attenuated [18], yet the effect persisted, implying that alternative signaling pathways may also contribute to RES-induced fiber-type remodeling in bovine myotubes. Importantly, AMPK and Akt/FoxO pathways exhibit functional cross-talk through shared downstream targets including PGC-1α and FoxO1 [19]. This interconnection provides a mechanistic rationale for investigating the Akt/FoxO axis as a complementary pathway when AMPK signaling is inhibited. The role of the Akt/FoxO pathway in regulating myofiber type shifting is unknown in oxidatively stressed bovine myotubes. Considering the pivotal involvement of the Akt/FoxO axis in both fiber-type plasticity and cellular antioxidant responses, this study used bovine myotube cells as the focus of the research. It explored the effects of oxidative stress on muscle fiber type composition and the intervention effect of RES. Additionally, the molecular mechanisms underlying these effects were investigated through the Akt/FoxO pathway.

While 400 μM H_2_O_2_ exceeds physiological ROS levels in healthy muscle tissue, it is relevant to pathological conditions such as ischemia-reperfusion injury, intense exercise-induced oxidative stress, or pre-slaughter stress in livestock, where localized H_2_O_2_ concentrations can reach micromolar levels [ 20 ]. This model thus represents a moderate-to-severe oxidative challenge rather than basal physiological conditions, allowing evaluation of RES protective effects under substantial oxidative stress.

## MATERIALS AND METHODS

### Cell culture

Cell culture was conducted as described previously [18] with slight modifications. Bovine myoblasts (catalog no. EY-K3208), derived from the skeletal muscle tissue of hindlimb muscles in two-month-old yellow cattle, were procured from Shanghai Yiyan Biotechnology. The cells were cultured under standard conditions: 37°C in a humidified incubator with 5% CO_2_, using Minimum Essential Medium (MEM; Solarbio) supplemented with 10% fetal bovine serum (FBS; BI) and a penicillin–streptomycin solution (100 IU/mL penicillin and 100 μg/mL streptomycin). For experimental plating, 10^4^ cells were seeded into each well of six-well plates. Once the cells reached about 70% confluence, the standard growth medium was substituted with a differentiation-inducing medium—comprising 98% MEM supplemented with 2% horse serum (Solarbio)—to promote myotube formation. The medium was refreshed every two days.

### Cell treatment

Upon achieving approximately 70% confluency, cultures were transitioned to differentiation medium for 72 h to induce differentiation. Cell differentiation and the completion of myotube formation were routinely confirmed by morphological observation under a phase-contrast microscope prior to subsequent treatments. The concentration of RES (10 μM) was selected based on our previous studies demonstrating its optimal efficacy in promoting slow-twitch fiber transformation in bovine myotubes without inducing cytotoxicity [18,21]. Then differentiation medium with or without RES (10 μM, purity≥99%; MCE) was changed to treat the cells for 24 h. Subsequently, the cells were treated with or without hydrogen peroxide (H_2_O_2_) (400 μM; Sigma-Aldrich) medium for 24 h to assess how oxidative challenge would alter skeletal-muscle fiber profiles and to evaluate the protective effect by RES.

To elucidate the molecular mechanism by which RES influences muscle fiber type transformation in oxidative-stressed bovine myotubes via the Akt/FoxO pathway, myotubes differentiated for 3 days were treated with SC79 (Akt activator, 10 μM; Selleck). The cells were divided in five groups: (i) control group, (ii) H_2_O_2_ group, (iii) SC79 group, (iv) H_2_O_2_+SC79 group, (v) H_2_O_2_+SC79+RES group (n = 4/group).

### Metabolic and antioxidant enzyme activities and malondialdehyde content assay

Cell lysates were utilized to assess the enzyme activities and malondialdehyde (MDA) content. The total protein concentration was measured using the bicinchoninic acid (BCA) protein assay kit (Nanjing Jiancheng Bioengineering Institute), in accordance with the manufacturer’s guidelines. Enzymatic activities of succinate dehydrogenase (SDH), malate dehydrogenase (MDH), lactate dehydrogenase (LDH), superoxide dismutase (SOD), glutathione peroxidase (GSH-Px), and catalase (CAT), along with MDA levels, were quantified using commercial colorimetric assay kits (Nanjing Jiancheng Bioengineering Institute), strictly adhering to the supplier’s protocol. Enzyme activity was expressed as units per milligram of protein (U/mg protein).

### RNA isolation and real-time quantitative polymerase chain reaction

Total RNA was extracted from bovine myotubes using RNAiso Plus (AG). The integrity of the RNA was confirmed through gel electrophoresis, and its purity was assessed using a NanoPhotometer (Implen) by measuring the absorbance ratio at 260/280 nm. All samples exhibited ratios ranging from 1.8 to 2.0, suggesting negligible contamination. Complementary DNA (cDNA) was synthesized from the RNA using the Evo M-MLV RT kit (AG) following the manufacturer’s instructions. Real-time quantitative polymerase chain reaction (PCR) was carried out on a CFX-96 Real-Time PCR system (Bio-Rad) with 2× SYBR Premix. The primers, designed using Beacon Designer software, are detailed in Supplement 1. GAPDH was used as the internal control, and the relative expression levels were calculated using the 2^−ΔΔCT^ method [22].

### Western blot analysis

Total protein was isolated from differentiated bovine myotubes using RIPA lysis buffer (Beyotime Biotechnology), supplemented with 2% phosphatase inhibitor cocktail and 1 mM phenylmethanesulfonyl fluoride (PMSF) to prevent proteolytic and dephosphorylation degradation. The cells were incubated on ice for 20 min directly in the culture plates, and the lysates were then centrifuged at 14,000×g for 10 min at 4°C to collect the supernatant. Protein content was quantified as previously outlined. Thirty-microgram aliquots were introduced onto an 8% resolving gel layered beneath a 5% stacking gel, electrophoresis commenced at 90 V for half an hour and was then ramped to 120 V for an additional sixty minutes. After electrophoretic separation, proteins were transferred onto polyvinylidene difluoride (PVDF) membranes at 90 V for 90 minutes while kept on ice. The membranes were then blocked with 5% bovine serum albumin (BSA) in 1×TBST (TBS containing 0.1% Tween-20) at room temperature for 1.5 hours. Following this, they were incubated overnight at 4°C with the following primary antibodies: slow MyHC (1:1,000; Sigma-Aldrich, M8421), fast MyHC (1:1,000; Sigma-Aldrich, M4276), Phosphatidylinositol-3-kinase (PI3K) (1:1,000; Cell Signaling Technology, 4249), p-Akt (Ser473) (1:1,000; Cell Signaling Technology, 4060), Akt (1:1,000; Cell Signaling Technology, 4691), p-FoxO1 (Ser256) (1:1,000; Cell Signaling Technology, 9461), FoxO1 (1:1,000; Cell Signaling Technology, 9454), p-FoxO3 (Ser253) (1:1,000; Cell Signaling Technology, 9466), FoxO3 (1:1,000; Cell Signaling Technology, 2497), and β-Actin (1:1,000; Abcam, ab8226). Subsequently, the blots underwent five washes with TBST before a 90-minute exposure at ambient temperature to horseradish-peroxidase-conjugated goat anti-rabbit IgG (1:5,000; Abcam, ab6721) and rabbit anti-mouse IgG (1:5,000; Abcam, ab6728). Immunoreactive bands were developed with enhanced chemiluminescence reagents and captured using a ChemiDoc MP imager (Bio-Rad). Densitometric quantification was performed with Image Lab software (v5.1; Bio-Rad). To ensure the reliability of the relative quantification, equal total protein loading was strictly verified using the BCA protein assay. Furthermore, preliminary quantitative analysis of the immunoblots confirmed that the expression level of β-Actin remained stable and was not significantly altered by H_2_O_2_, RES, or SC79 treatments across all experimental groups (p>0.05), validating its suitability as an internal loading control in this study.

### Statistical analysis

Data processing relied on version 26.0 of the SPSS platform (IBM, Chicago, IL, USA). All experiments were performed with four independent biological replicates (n = 4/group). Myofiber-type distribution, transcript levels, protein abundance, and enzyme activity values were analyzed by one-way ANOVA, multiple comparisons were subsequently evaluated with LSD test. Given the relatively small sample size per group (n = 4), we employed the Bonferroni post hoc test to minimize the risk of Type I error during multiple comparisons, after confirming that all datasets met the assumptions of normality and homogeneity of variances. Significance was set at p<0.05. Data were presented as means±SEM. Graphs were prepared with Origin 2022.

## RESULTS AND DISCUSSION

**Figure 1:**
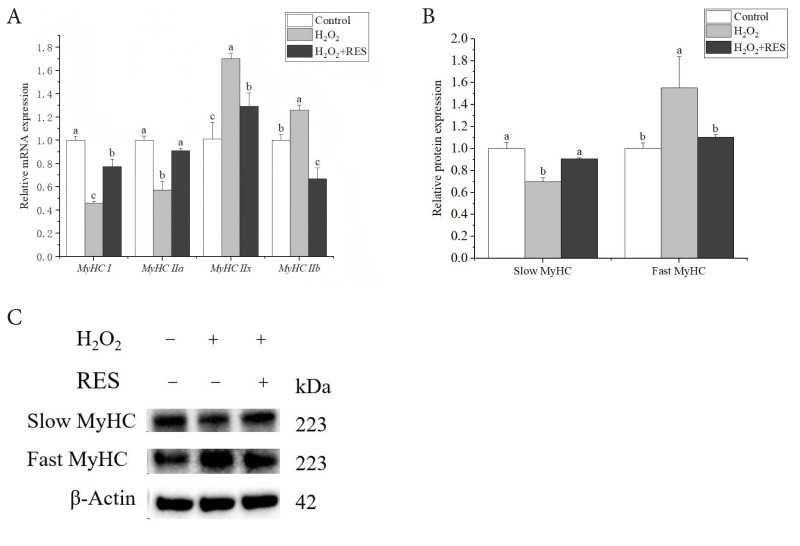
Effects of oxidative stress and resveratrol (RES) on muscle fiber type-related gene and protein expression in bovine myotubes. (A) Relative mRNA expression of MyHC I, MyHC IIa, MyHC IIx, and MyHC IIb. (B) Relative protein expression of slow MyHC and fast MyHC. (C) Representative Western blot images of slow MyHC and fast MyHC. ^a–c^ Different superscript letters indicate significant differences (p<0.05). H_2_O_2_, hydrogen peroxide; RES, resveratrol; MyHC I, MyHC IIa, MyHC IIx, MyHC IIb, myosin heavy chain isoforms representing different muscle fiber types; Slow MyHC, Fast MyHC, protein expression levels of slow and fast myosin heavy chains, respectively; kDa, kilodalton; β-Actin, loading control for Western blotting.

To evaluate how oxidative challenge and RES modulate myofiber phenotypes in bovine myotubes, the genes and proteins expression level were determined, respectively. As shown in Figure 1A, oxidative stress significantly decreased the mRNA expression levels of *MyHC* I and *MyHC* IIa in bovine myotubes, and the RES treatment group increased the mRNA expression levels of *MyHC* I and *MyHC* IIa compared with the oxidative stress group (p<0.05). Compared with the control group, oxidative stress increased the mRNA expression levels of *MyHC* IIx and *MyHC* IIb (p<0.05), and RES decreased the mRNA expression levels of *MyHC* IIx and *MyHC* IIb in bovine myotubes under oxidative stress conditions (p<0.05). As depicted in Figures 1B, 1C, slow MyHC was markedly suppressed, whereas the fast MyHC was elevated, following exposure to ROS (p<0.05). This shift was counteracted by RES, such that the slow MyHC was restored and the fast MyHC was attenuated relative to the oxidatively challenged controls (p<0.05). This indicated that oxidative stress caused the muscle fiber types of bovine myotubes to be transformed from slow to fast, and RES could attenuate the effect of oxidative stress on muscle fiber types.

Earlier work from our laboratory demonstrated that supplementing beef cattle diets with RES shifted the fiber-type profile toward oxidative type I fibers in the *longissimus thoracis*, *psoas major*, and *semitendinosus* muscles [21]. In bovine myotubes, RES elevated the transcript abundance of oxidative fibers (*MyHC I* and *IIa*) and concurrently augmented the abundance of the slow MyHC protein [18]. Oxidative stress significantly decreased the protein expression of slow muscle fiber, whereas the addition of the antioxidant naringenin, a natural polyphenol, significantly increased slow muscle protein expression [23], similar to the results of the present study.

**Figure 2:**
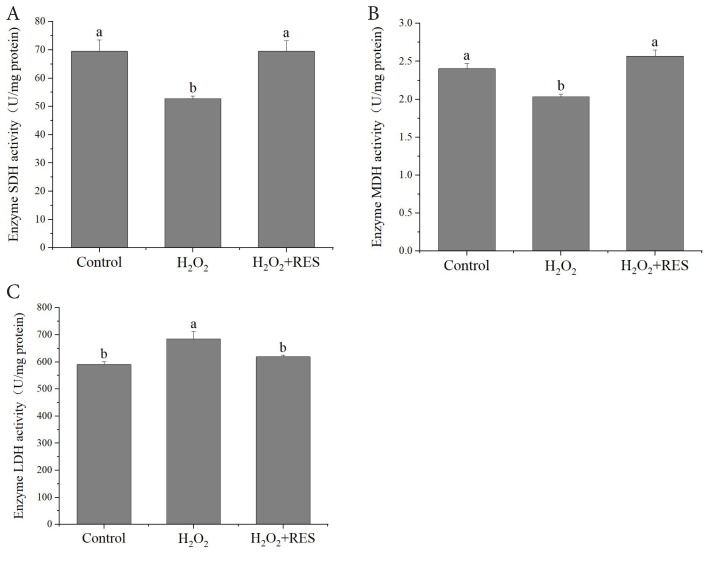
Effects of oxidative stress and resveratrol (RES) on metabolic enzyme activities in bovine myotubes. (A) Enzyme activity of succinic dehydrogenase (SDH). (B) Enzyme activity of malate dehydrogenase (MDH). (C) Enzyme activity of lactate dehydrogenase (LDH). ^a,b^ Different superscript letters indicate significant differences (p<0.05) between groups. SDH, succinate dehydrogenase (indicative of oxidative metabolism); H_2_O_2_, hydrogen peroxide; RES, resveratrol; MDH, malate dehydrogenase (also indicative of oxidative metabolism); LDH, lactate dehydrogenase (indicative of anaerobic glycolysis).

**Figure 3:**
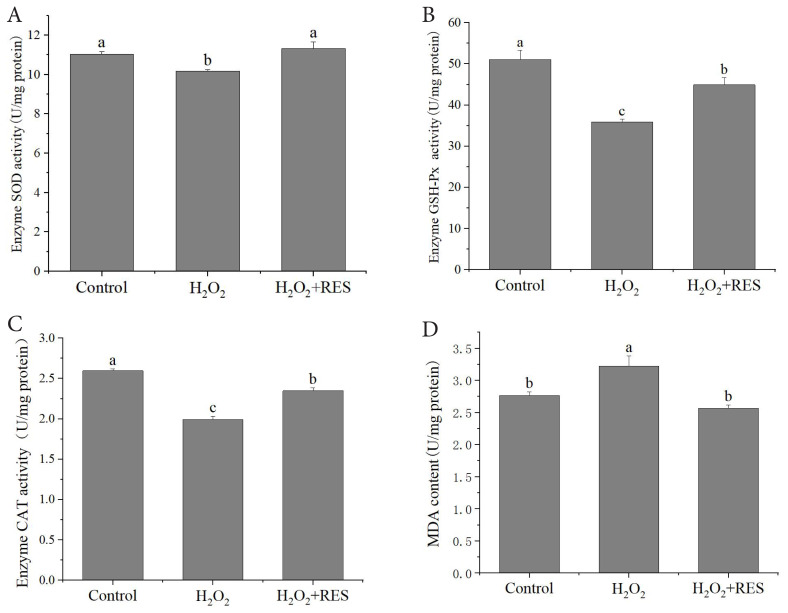
Effects of oxidative stress and resveratrol (RES) on antioxidant enzyme activities and malondialdehyde (MDA) content in bovine myotubes. (A) Enzyme activity of superoxide dismutase (SOD). (B) Enzyme activity of glutathione peroxidase (GSH-Px). (C) Enzyme activity of catalase (CAT). (D) Malondialdehyde (MDA) content. ^a–c^ Different superscript letters indicate significant differences (p<0.05) between groups. SOD, superoxide dismutase; H_2_O_2_, hydrogen peroxide; RES, resveratrol; GSH-Px, glutathione peroxidase; CAT, catalase; MDA, malondialdehyde (a product of lipid peroxidation).

Metabolic enzyme activities varied across fiber phenotypes. Therefore, profiling these enzyme activities provided an indirect indicator of alterations in the fiber type distribution. In general, SDH and MDH activities were higher in oxidative muscle fibers, whereas LDH activity was higher in glycolytic muscle fibers [24]. As illustrated in Figures 2A, 2B, the activities of both SDH and MDH were markedly attenuated under oxidative conditions. These enzymatic levels were subsequently restored by RES supplementation in bovine myotubes relative to the oxidatively challenged controls (p<0.05). As shown in Figure 2C, oxidative stress increased LDH activity in bovine myotubes, which was subsequently reduced by RES treatment (p<0.05). Collectively, the findings implied that oxidative stress shifted the fiber profile toward a glycolytic phenotype, while RES treatment counterbalanced this transition in cultured bovine myotubes. Oxidative stress causes an imbalance in the cellular redox system, leading to a decrease in antioxidant enzyme activities and an increase in lipid oxidation products [25]. Figures 3A–3C revealed that, upon oxidative stress challenge, the enzymatic activities of SOD, GSH-Px, and CAT in cultured bovine myotubes were substantially diminished (p<0.05), after adding RES, the result was completely opposite. As shown in Figure 3D, oxidative stress increased the content of MDA in bovine myotubes (p<0.05), and RES decreased the levels of MDA in bovine myotubes under oxidative stress conditions (p<0.05). The results indicated that RES could effectively protect bovine myotubes from oxidative damage caused by H_2_O_2_. RES can balance the cellular redox state by enhancing the activities of various antioxidant defense enzymes, thus protecting cells from oxidative damage [26].

Compared to glycolytic fibers, oxidative myofibers (types I and IIa) were rich with mitochondria and enzymes that drove oxidative metabolism. Given that SDH and MDH functioned as key aerobic respiration enzyme in mitochondria, oxidative fiber types displayed markedly greater activities of these enzymes. Whereas glycolytic muscle fibers (type IIb) were known to contain fewer mitochondria and exhibited higher glycolytic enzyme activity, LDH, a glycolytic enzyme involved in the glycolytic pathway, had been recognized for its higher activity in glycolytic muscle fibers [27]. These characteristics had been well established in the literature prior to these studies. The present findings indicated that exposure to oxidative stress markedly suppressed the enzymatic function of both SDH and MDH, while simultaneously elevating LDH activity, and RES ameliorated the above effects caused by oxidative stress. It suggested that oxidative stress might have affected energy metabolism, which in turn affected muscle fiber type composition. Oxidative stress increased cardiac LDH activity in rats, whereas ellagic acid treatment reduced an oxidative stress-induced increase in LDH activity [28]. Oxidative stress decreased the activity of SDH, MDH in rats, while rats fed betanin nanoparticles before oxidative stress exhibited increased the activity of SDH, MDH [29]. Findings reported earlier corroborated the outcomes observed in this investigation. Redox homeostasis collapsed under oxidative stress, whereas RES conferred cytoprotection by mitigating the resultant oxidative injury [30]. RES might also have attenuated the effects of oxidative stress on muscle fiber type by reducing cellular oxidative stress, and the results showed that oxidative stress decreased the activities of bovine myotubes antioxidant enzymes SOD, GSH-Px and CAT, and increased the lipid peroxide MDA content, while RES pretreatment attenuated the above enzyme activities caused by oxidative stress changes (Figure 3). Similar results were obtained when studying the protection of RES against H_2_O_2_-induced oxidative stress in bovine skeletal muscle cells [15].

**Figure 4:**
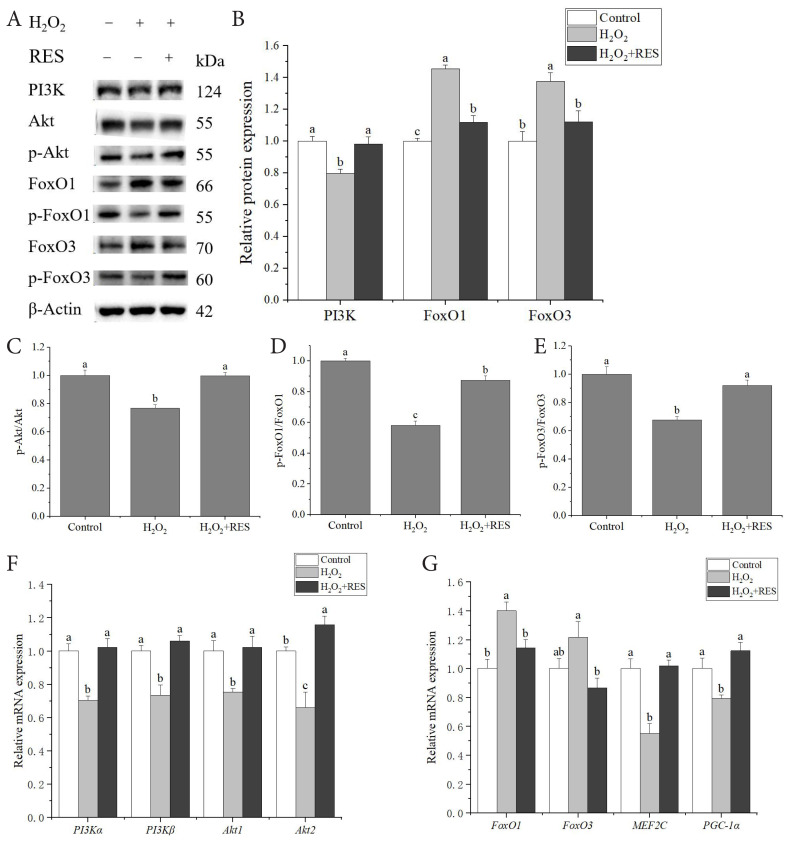
Effects of oxidative stress and resveratrol (RES) on the Akt/FoxO pathway and mitochondrial biogenesis in bovine myotubes. (A) Representative Western blot images of PI3K, Akt, p-Akt, FoxO1, p-FoxO1, FoxO3, and p-FoxO3. (B) Relative protein expression of PI3K, FoxO1, and FoxO3. (C) Ratio of p-Akt to total Akt (p-Akt/Akt). (D) Ratio of p-FoxO1 to total FoxO1 (p-FoxO1/FoxO1). (E) Ratio of p-FoxO3 to total FoxO3 (p-FoxO3/FoxO3). (F) Relative mRNA expression of PI3Kα, PI3Kβ, Akt1, and Akt2. (G) Relative mRNA expression of FoxO1, FoxO3, MEF2C, and PGC-1α. ^a–c^ Different letters indicate significant differences (p<0.05) between groups. H_2_O_2_, hydrogen peroxide; RES, resveratrol; PI3K, phosphoinositide 3-kinase; p-Akt/Akt, p-FoxO1/FoxO1, and p-FoxO3/FoxO3, ratios of phosphorylated to total protein reflecting activation status; FoxO1/FoxO3, forkhead box transcription factors O1 and O3; MEF2C, myocyte enhancer factor 2C; PGC-1α, peroxisome proliferator-activated receptor gamma coactivator 1-alpha.

The above data demonstrated that oxidative challenge accelerates the shift from slow-to fast-twitch phenotypes in bovine myotubes, however, the involvement of the Akt/FoxO axis in mediating this transition and its precise molecular basis remained unresolved. To investigate whether oxidative stress promoted muscle fiber type transformation through the Akt/FoxO signaling pathway, Akt/FoxO pathway-related genes and protein expression were examined. As shown in Figures 4A–4E, Western blot analysis revealed that H_2_O_2_ treatment significantly altered the expression levels of Akt/FoxO pathway-related proteins compared to the control group. Specifically, oxidative stress significantly upregulated the protein expression of total FoxO1 and total FoxO3 (increasing to 1.45-fold and 1.38-fold of the control, respectively, p<0.05). Concurrently, H_2_O_2_ treatment markedly inhibited the expression or activation of upstream signaling molecules: the PI3K catalytic subunit was downregulated, and the ratio of phosphorylated Akt (p-Akt) to total Akt was reduced by 23% (p-Akt/Akt ratio decreased to 0.77, p<0.05). Correspondingly, the phosphorylation levels of its downstream substrates were also significantly decreased, with the p-FoxO1/FoxO1 and p-FoxO3/FoxO3 ratios dropping to 0.58-fold and 0.68-fold of the control, respectively (p<0.05). Upon treatment with RES, these H_2_O_2_-induced changes were significantly ameliorated (p-Akt/Akt, p-FoxO1/FoxO1, and p-FoxO3/FoxO3 ratios recovered to 1.0, 0.88, and 0.92, respectively, and total FoxO1/3 expression also decreased), indicating that RES effectively counteracts the disruption of the Akt/FoxO pathway caused by oxidative stress. As shown in Figures 4F, 4G, oxidative stress decreased the mRNA expression levels of *PI3Kα*, *PI3Kβ, Akt1, Akt2, MEF2C*, and *PGC-1α* in bovine myotubes and increased the mRNA expression of *FoxO1* (p<0.05). All these oxidative stress effects on bovine myotubes were attenuated by the addition of RES. It suggested that oxidative stress might have reduced FoxO nucleoplasmic shuttling by inhibiting Akt phosphorylation, which resulted in lower levels of FoxO phosphorylation and increased FoxO expression. Taken together, oxidative stress might regulate muscle fiber types conversion by inhibiting the Akt/FoxO signaling pathway, and RES attenuated the inhibitory effect of oxidative stress on this pathway.

**Figure 5:**
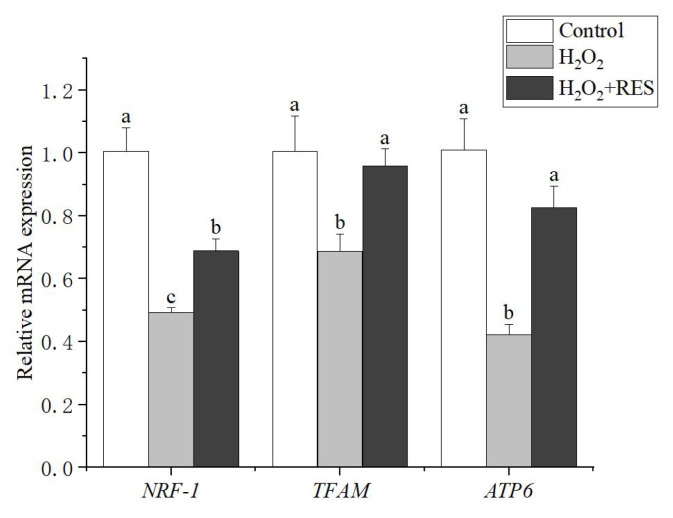
Effects of oxidative stress and resveratrol (RES) on the mRNA expression of mitochondrial biogenesis-related genes in bovine myotubes. ^a–c^ Different letters indicate significant differences (p<0.05) between groups. H_2_O_2_, hydrogen peroxide; RES, resveratrol; NRF1, nuclear respiratory factor 1; TFAM, mitochondrial transcription factor A; ATP6, ATP synthase F0 subunit 6.

ROS compromised the integrity of skeletal muscle mitochondria and provoked a decline in oxidative phosphorylation efficiency. To evaluate how oxidative challenge influenced mitochondrial biogenesis in bovine myotubes and to assess RES-mediated protection against oxidative damage, quantitative PCR was used to quantify transcript levels of key regulators—*NRF-1*, *TFAM*, and *ATP6*—involved in the expansion of the mitochondrial network. The result indicated that oxidative stress decreased the gene expression levels of *NRF-1, TFAM* and *ATP6*, and compared with the oxidative stress group, the RES pretreatment group increased the gene expression levels of *NRF-1, TFAM* and *ATP6* (p<0.05, Figure 5). This indicated that oxidative stress suppresses the transcriptional activity of genes involved in mitochondrial biogenesis, and RES could effectively protect cellular mitochondrial synthesis from oxidative stress damage. Exposure of HepG2 cells to dimethoxyethyl phthalate elicited an oxidative insult that diminished ATP6 transcript abundance and concurrently attenuated the abundance of *NRF-1* and *TFAM* proteins [31]. The above studies were consistent with the results of the present study.

The Akt/FoxO axis governed the phenotypic shift between myofiber classes, dietary lycopene drove mice skeletal muscle toward a slower-twitch profile by engaging this Akt/FoxO1 cascade [32]. Likewise, leucine prompted porcine myofibers to shift from fast-twitch toward a slow-twitch identity via modulation of the Akt/FoxO1 cascade [33]. It was shown that RES activated Akt activity in rat bone marrow mesenchymal stem cells, mouse osteoblasts, and rat myocardium. Akt phosphorylates FoxO1 and thus affected its transcriptional activity, while some studies found that RES inhibited the expression of FoxO1 in mouse C2C12 cells and FoxO3 in mouse lung tissues [34], suggesting that RES could activate the Akt/FoxO pathway and thus inhibit the expression of FoxO, which was in line with the present findings.

**Figure 6:**
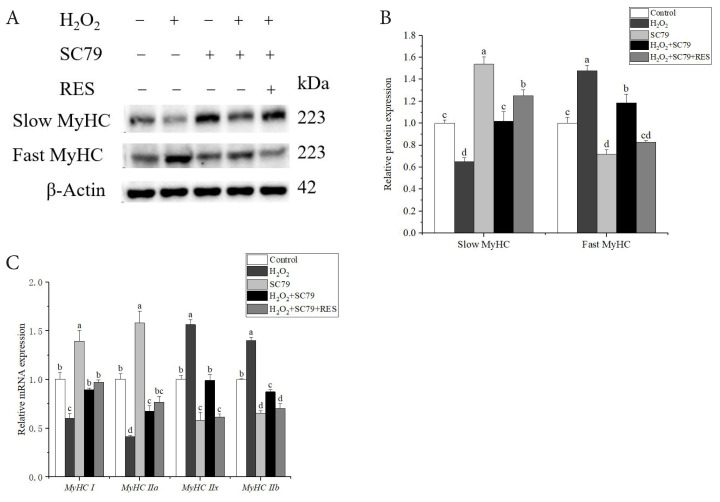
Effects of the Akt activator SC79 and RES on muscle fiber type transition in bovine myotubes under oxidative stress. (A) Representative Western blot images of slow MyHC and fast MyHC. (B) Relative protein expression of slow MyHC and fast MyHC. (C) Relative mRNA expression of MyHC I, MyHC IIa, MyHC IIx, and MyHC IIb. ^a–d^ Different letters indicate significant differences (p<0.05) between groups. H_2_O_2_, hydrogen peroxide; SC79, Akt activator; RES, resveratrol; Slow MyHC, slow myosin heavy chain; Fast MyHC, fast myosin heavy chain; β-Actin, loading control; MyHC I, MyHC IIa, MyHC IIx, MyHC IIb, myosin heavy chain isoforms.

To determine whether the Akt/FoxO axis mediates oxidative-stress-induced fiber-type switching, bovine myotubes were exposed to the Akt activator SC79 to enhance kinase activity and phosphorylation status, followed by profiling the transcripts and proteins governing myofiber phenotype. As shown in Figures 6A, 6B, SC79 attenuated the decrease in slow MyHC protein expression and increase in fast MyHC protein expression caused by oxidative stress compared with the oxidative stress group (p<0.05). The addition of RES further attenuated the effect of oxidative stress on muscle fiber type composition (p<0.05). As shown in Figure 6C, the down-regulation of *MyHC I* and *MyHC IIa* mRNA expression in bovine myotubes by oxidative stress was attenuated by the addition of SC79, while the up-regulation of *MyHC IIx* and *MyHC IIb* mRNA expression by oxidative stress was weakened by the addition of SC79 (p<0.05). Furthermore, the addition of RES synergistically enhanced SC79’s attenuation of the oxidative stress-induced alterations in muscle fiber types.

**Figure 7:**
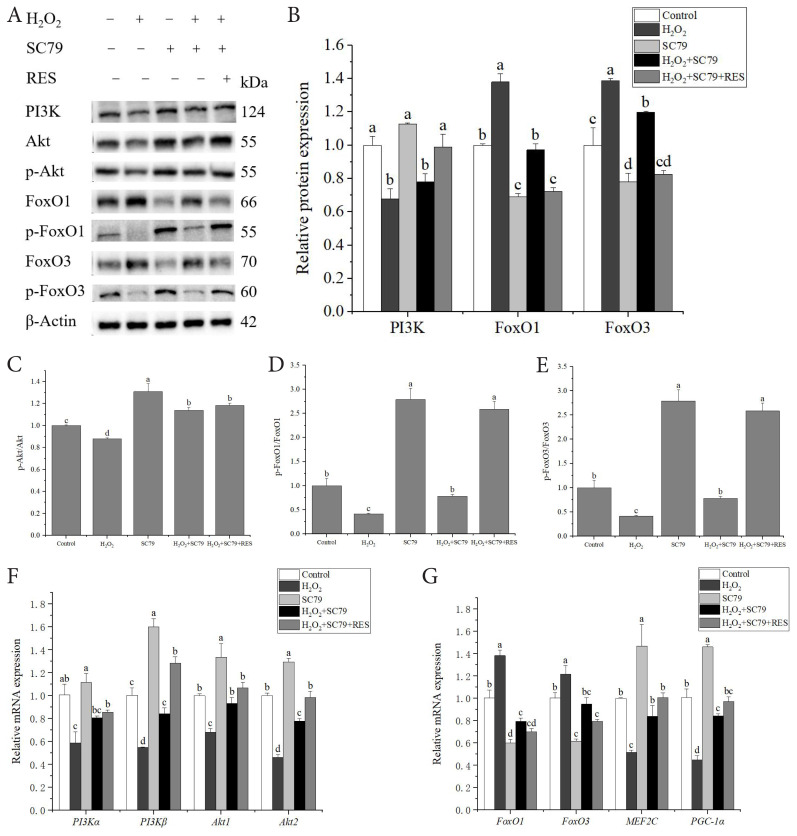
Effects of the Akt activator SC79 and resveratrol (RES) on Akt/FoxO signaling pathway and muscle fiber type switching in bovine myotubes under oxidative stress. (A) Representative Western blot images of PI3K, Akt, p-Akt, FoxO1, p-FoxO1, FoxO3, and p-FoxO3. (B) Relative protein expression of PI3K, FoxO1, and FoxO3. (C) Ratio of p-Akt to total Akt (p-Akt/Akt). (D) Ratio of p-FoxO1 to total FoxO1 (p-FoxO1/FoxO1). (E) Ratio of p-FoxO3 to total FoxO3 (p-FoxO3/FoxO3). (F) Relative mRNA expression of PI3Kα, PI3Kβ, Akt1, and Akt2. (G) Relative mRNA expression of FoxO1, FoxO3, MEF2C, and PGC-1α. ^a–d^ Different letters indicate significant differences (p<0.05) between groups. H_2_O_2_, hydrogen peroxide; SC79, Akt activator; PI3K, phosphoinositide 3-kinase; p-Akt/Akt, p-FoxO1/FoxO1, and p-FoxO3/FoxO3, ratios of phosphorylated to total protein reflecting activation status; FoxO1/FoxO3, forkhead box transcription factors O1 and O3; MEF2C, myocyte enhancer factor 2C; PGC-1α, peroxisome proliferator-activated receptor gamma coactivator 1-alpha.

Additional experiments were therefore designed to verify whether oxidative challenge drives the shift in myofiber phenotype via the Akt/FoxO cascade. Akt/FoxO signaling pathway-related signaling molecule gene and protein expression was detected in the presence of Akt activator SC79. As shown in Figures 7A, 7B, compared with the oxidative stress group, SC79 significantly attenuated the promoting effect of oxidative stress on the protein expression of FoxO1 and FoxO3 in bovine myotubes. Figures 7C–7E showed that SC79 significantly counteracted the inhibitory effect of oxidative stress on the protein expression of p-Akt/Akt, p-FoxO1/FoxO1 and p-FoxO3/FoxO3 (p<0.05), the addition of RES further synergized SC79 to attenuate the effects of oxidative stress on the above protein expressions. As shown in Figures 7F, 7G, SC79 treatment significantly attenuated the inhibitory effects of oxidative stress on *PI3Kβ*, *Akt1*, *Akt2* and *PGC-1α* mRNA expression (p<0.05), whereas the attenuation of *PI3Kα* and *MEF2C* was not statistically significant (*PI3Kα*: p = 0.0918; *MEF2C*: p = 0.1405). Meanwhile, SC79 counteracted the promoting effect of oxidative stress on *FoxO1* and *FoxO3* mRNA expression (p<0.05). In addition, the addition of RES further synergized with SC79 to attenuate the effect of oxidative stress on the expression of the above genes. This suggested that oxidative stress affected muscle fiber type conversion by inhibiting the Akt/FoxO signaling pathway, and RES might effectively ameliorate the effects of oxidative stress by activating this pathway.

**Figure 8:**
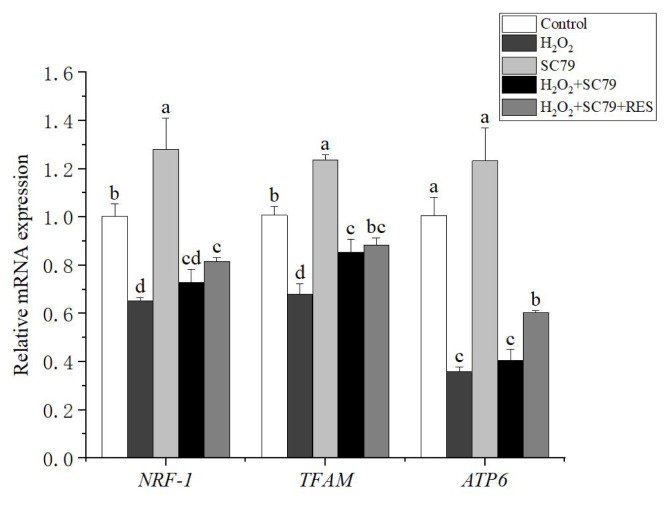
Effects of the Akt activator SC79 and resveratrol (RES) on the mRNA expression of mitochondrial biogenesis-related genes in bovine myotubes under oxidative stress. ^a–d^ Different letters indicate significant differences (p<0.05) between groups. H_2_O_2_, hydrogen peroxide; SC79, Akt activator; NRF-1, nuclear respiratory factor 1; TFAM, mitochondrial transcription factor A; ATP6, ATP synthase F0 subunit 6.

To investigate the effect of activating Akt/FoxO on mitochondrial biogenesis in bovine myotubes, the expression of mitochondrial synthesis-related genes was examined. As shown in Figure 8, the Akt-specific activator SC79 significantly counteracted the oxidative stress-induced suppression of *TFAM* mRNA expression (p<0.05). Additionally, SC79 treatment resulted in numerically higher *NRF-1* and *ATP6* mRNA levels compared to the oxidative stress group, though these differences did not reach statistical significance (*NRF-1*: p = 0.0954; *ATP6*: p = 0.1096). Similarly, RES attenuated the inhibitory effects of oxidative stress on *TFAM*, *NRF-1*, and *ATP6* mRNA expression. These findings implied that oxidative stress inhibited mitochondrial biogenesis to a certain extent by suppressing the Akt/FoxO signaling pathway, which in turn affected the transformation of bovine myotube muscle fiber types. Covalent processing—through phosphoryl, acetyl, ubiquitin, methyl, or glycosyl adducts—modulated FoxO function by dictating its catalytic state and intracellular trafficking [35]. Phosphorylation was the most important post-translational modification of FoxO proteins, which could be induced by a variety of protein kinases, and different enzymes regulated phosphorylation at different locations in FoxO proteins to produce different biological effects, mainly including Akt, AMPK and SGK1, etc. The phosphorylated nuclear/cytoplasmic shuttling of FoxO dependent on phosphorylation was mainly regulated by Akt, and the phosphorylation of FoxO by Akt led to FoxO being cytoplasm to be isolated and unable to initiate downstream target genes related to muscle fiber type regulation, which in turn inhibited the transcription of target genes. PI3K was an upstream regulator of Akt, and activation of the PI3K/Akt pathway promoted the phosphorylation of FoxO proteins [36].

To verify that oxidative stress regulates muscle fiber type transformation through inhibition of the Akt/FoxO signaling pathway, cells were treated with the addition of the Akt activator SC79. The results showed that SC79 attenuated the oxidative stress-induced decrease in slow MyHC protein expression and an increase in fast MyHC protein expression, and eliminated the oxidative stress-induced down-regulation of *MyHC I* and *MyHC IIa* mRNA expression as well as the up-regulation of *MyHC IIx* and *MyHC IIb* mRNA expression, and that the effect of oxidative stress on the composition of muscle fiber types was further attenuated by the addition of RES. Addition of SC79 activated Akt and increased the level of Akt phosphorylation, and decreased the expression of FoxO1 and FoxO3 under oxidative stress, which was consistent with previous findings that SC79 promotes Akt phosphorylation in mouse brain. This suggested that oxidative stress was responsible for the conversion of muscle fiber type from slow to fast muscle by inhibiting the Akt/FoxO signaling pathway, and that RES might partially counteract the effects of oxidative stress by activating the Akt/FoxO signaling pathway. In addition, we found that activation of Akt activity increased *TFAM* gene expression, suggesting that the Akt/FoxO signaling pathway may also have a relationship with mitochondrial synthesis.

## CONCLUSION

This study demonstrates that oxidative stress induces a slow-to-fast myofiber shift in bovine myotubes by suppressing the Akt/FoxO signaling pathway and mitochondrial biogenesis. RES effectively counteracts this shift by reactivating the Akt/FoxO axis, enhancing antioxidant defense, and improving mitochondrial function.

PGC-1α was a coactivator of FoxO1 [37]. PGC-1α had a role in differentiating type I muscle fibers and promoted the conversion of glycolytic to oxidative muscle fiber types, a process in which FoxO1 might also be involved. FoxO1 and PGC-1α were found to interact in the insulin-regulated gluconeogenic pathway in mice, and the FoxO1-mediated reduction of type I muscle fibers might be due to the fact that FoxO1 proteins inhibited some of the functions of PGC-1α by interacting with PGC-1α proteins. In the present study, oxidative stress led to a decrease in type I muscle fiber expression, along with a significant increase in FoxO1 gene and protein expression and downregulation of PGC-1α expression. This might be due to the fact that FoxO1 inhibited some of the functions of PGC-1α thereby decreasing slow muscle expression and increasing fast muscle expression. However, further studies are warranted to confirm this interaction, as the present data demonstrate an observational rather than causal relationship.

Preferentially expressed in oxidative, slow-contracting fibers, the transcription factor MEF2C exerted pivotal control over myogenesis [38]. MEF2C overexpression in mice drives fast-to-slow fiber conversion via calcium signaling, boosting oxidative fibers and endurance. Dietary supplementation with sodium butyrate promoted the formation of oxidized fibers and upregulated the expression of phosphorylated FoxO1 and MEF2C proteins in mice, suggesting that the transformation of muscle fiber types might be regulated by inhibiting FoxO1 and upregulating the expression of MEF2C [39]. In the skeletal muscles of FoxO1 transgenic mice, the expression levels of both MEF2C and CaMK were significantly reduced, suggesting that FoxO1 might regulate the expression of type I muscle fiber genes by inhibiting MEF2C and CaMK expression and thus regulating the expression of type I muscle fiber genes [40]. Similarly, oxidative stress in the present study led to decreased MEF2C expression alongside elevated FoxO1 expression. As both MEF2C and CaMK were reduced in FoxO1 transgenic mice, the observed MEF2C down-regulation may involve coordinated regulation of multiple signaling components rather than direct FoxO1-mediated transcriptional repression.

The implications of this work span both basic muscle biology and practical agricultural application. Theoretically, our findings confirm that the Akt/FoxO pathway is essential for mediating fiber-type transitions triggered by oxidative stress in bovine muscle. By mapping the protective mechanisms of RES, this study broadens our understanding of the complex networks regulating myofiber plasticity. From an applied perspective, these results point toward a viable strategy for maintaining meat quality. It is important to note, however, that these experiments were restricted to an *in vitro* bovine myotube model. Although peri-slaughter oxidative stress is a well-known catalyst for meat quality deterioration, the current research lacks *in vivo* validation, structural muscle assessments, and direct meat quality evaluations. Nevertheless, because fiber-type composition directly influences meat traits, utilizing RES or comparable antioxidants to prevent detrimental fiber-type shifts remains a highly plausible intervention. Ultimately, this mechanistic evidence supports the development of targeted nutritional strategies to optimize key commercial attributes, such as tenderness and water-holding capacity.

A few methodological limitations must be considered when interpreting these results. While the SC79 treatment successfully rescued the oxidative phenotype, establishing definitive causality within the Akt/FoxO pathway requires further validation using targeted inhibitors or FoxO1/3 genetic modification. Additionally, the precise transcriptional control exerted by FoxO—specifically its nuclear translocation and direct binding to *MyHC* promoters—needs confirmation through ChIP assays. Finally, our assessment of mitochondrial biogenesis relied on mRNA transcripts. To fully substantiate these transcriptomic trends, subsequent research must include direct physiological quantification, such as measuring mtDNA copy numbers or performing structural evaluations via electron microscopy.

In conclusion, RES alleviates oxidative stress-induced fast-twitch fiber transformation in bovine myotubes primarily by activating the Akt/FoxO signaling pathway. In summary, detailing this mechanism provides a concrete biological rationale for meat quality optimization. Since *in vitro* models inherently lack systemic variables—such as RES bioavailability and fluctuating metabolic stress—these foundational results must now be tested *in vivo*. Moving forward, live-animal studies are essential to verify efficacy and establish practical dosing strategies before integrating such interventions into standard livestock management. And it is necessary to further investigate the actual effects of fiber - type changes on meat quality parameters like tenderness and water - holding capacity in these animal models.

## Data Availability

Upon reasonable request, the datasets of this study can be available from the corresponding author.

## SUPPLEMENTARY MATERIAL

**Supplement 1.** Primers used for real-time quantitative PCR.

## References

[1] Li Y, Fang J, Jiao Y (2025). MiR-21-5p enhances differentiation and mitigates oleic acid-induced lipid droplet accumulation in C2C12 myoblasts by targeting FBXO11. Anim Biosci.

[2] Pette D, Staron RS (2000). Myosin isoforms, muscle fiber types, and transitions. Microsc Res Tech.

[3] Guo X, Xu S, Fu C, Meng X (2025). L-lysine improves pork quality during postmortem aging: insights into meat quality, protein properties, and enzyme activities. Anim Biosci.

[4] Listrat A, Gagaoua M, Normand J (2020). Contribution of connective tissue components, muscle fibres and marbling to beef tenderness variability in longissimus thoracis, rectus abdominis, semimembranosus and semitendinosus muscles. J Sci Food Agric.

[5] Lu X, Yang Y, Zhang Y (2021). The relationship between myofiber characteristics and meat quality of Chinese Qinchuan and Luxi cattle. Anim Biosci.

[6] Hou X, Niu N, Liu X, Wang L, Wang L, Zhang L (2025). Dynamic changes in chromatin accessibility and gene expression involved in fetal myogenesis of Min pigs. Anim Biosci.

[7] Lushchak VI (2011). Environmentally induced oxidative stress in aquatic animals. Aquat Toxicol.

[8] Eijkelenboom A, Burgering BMT (2013). FOXOs: signalling integrators for homeostasis maintenance. Nat Rev Mol Cell Biol.

[9] Ji LL, Yeo D, Kang C (2020). Muscle disuse atrophy caused by discord of intracellular signaling. Antioxid Redox Signal.

[10] Yuan Y, Shi X, Liu Y, Yang G (2011). FoxO1 regulates muscle fiber-type specification and inhibits calcineurin signaling during C2C12 myoblast differentiation. Mol Cell Biochem.

[11] Russell SJ, Schneider MF (2020). Alternative signaling pathways from IGF1 or insulin to AKT activation and FOXO1 nuclear efflux in adult skeletal muscle fibers. J Biol Chem.

[12] Dodd SL, Gagnon BJ, Senf SM, Hain BA, Judge AR (2010). Ros-mediated activation of NF-κB and Foxo during muscle disuse. Muscle Nerve.

[13] Powers SK, Schrager M (2022). Redox signaling regulates skeletal muscle remodeling in response to exercise and prolonged inactivity. Redox Biol.

[14] Xiong H, Zhang Y, Zhao Z (2024). Investigation of single nucleotide polymorphisms in differentially expressed genes and proteins reveals the genetic basis of skeletal muscle growth differences between Tibetan and large white pigs. Anim Biosci.

[15] Cheng K, Yu C, Li Z (2020). Resveratrol improves meat quality, muscular antioxidant capacity, lipid metabolism and fiber type composition of intrauterine growth retarded pigs. Meat Sci.

[16] Zhang C, Luo J, Yu B (2015). Dietary resveratrol supplementation improves meat quality of finishing pigs through changing muscle fiber characteristics and antioxidative status. Meat Sci.

[17] Liu M, Cheng L, Li X, Wang H, Wang M, Gan L (2023). Resveratrol reverses myogenic induction suppression caused by high glucose through activating the SIRT1/AKT/FOXO1 pathway. Nat Prod Commun.

[18] Zhang J, Li J, Liu Y (2023). Effect of resveratrol on skeletal slow-twitch muscle fiber expression via AMPK/PGC-1α signaling pathway in bovine myotubes. Meat Sci.

[19] Chen K, Gao P, Li Z (2022). Forkhead Box O signaling pathway in skeletal muscle atrophy. J Cachexia Sarcopenia Muscle.

[20] Powers SK, Ozdemir M, Hyatt H (2020). Redox control of proteolysis during inactivity-induced skeletal muscle atrophy. Antioxid Redox Signal.

[21] Sun J, Zuo H, Liu Y (2025). The study of resveratrol promotes the transformation of bovine muscle fiber types through Akt/FoxO signaling pathway. Food Ferment Ind.

[22] Livak KJ, Schmittgen TD (2001). Analysis of relative gene expression data using real-time quantitative PCR and the 2−ΔΔCT method. Methods.

[23] Shi Y, Wang Y, Shi X, Zhang X, Zhang S (2023). Naringenin promotes the expression of oxidized myofibers via the PKA signaling pathway in C57BL/6J mice and C2C12 cells. J Funct Foods.

[24] Takekura H, Yoshioka T (1990). Ultrastructural and metabolic characteristics of single muscle fibres belonging to the same type in various muscles in rats. J Muscle Res Cell Motil.

[25] Birben E, Sahiner UM, Sackesen C, Erzurum S, Kalayci O (2012). Oxidative stress and antioxidant defense. World Allergy Organ J.

[26] Truong VL, Jun M, Jeong WS (2018). Role of resveratrol in regulation of cellular defense systems against oxidative stress. BioFactors.

[27] Jia AF, Feng JH, Zhang MH (2015). Effects of immunological challenge induced by lipopolysaccharide on skeletal muscle fiber type conversion of piglets. J Anim Sci.

[28] Huang C, Cheng H, Wen B (2026). Effects of AMPK/PGC-1ced by lipopolysaccharide on skeletal muscle fiber type overwintering starvation and its molecular mechanism: review. Anim Biosci.

[29] Choi S, Shin S (2025). Inhibition of myotube formation by platelet-derived growth factor subunit B in QM7 cells. Anim Biosci.

[30] Fukui M, Choi HJ, Zhu BT (2010). Mechanism for the protective effect of resveratrol against oxidative stress-induced neuronal death. Free Radic Biol Med.

[31] Liu H, Zhu S, Han W, Cai Y, Liu C (2021). DMEP induces mitochondrial damage regulated by inhibiting Nrf2 and SIRT1/PGC-1α signaling pathways in HepG2 cells. Ecotoxicol Environ Saf.

[32] Wen W, Chen X, Huang Z (2021). Lycopene promotes a fast-to-slow fiber type transformation through Akt/FoxO1 signaling pathway and miR-22-3p. J Funct Foods.

[33] Wang Y, Zhang D, Liu Y (2024). Research progress on the regulating factors of muscle fiber heterogeneity in livestock: a review. Animals.

[34] Singh N, Nagar E, Gautam A, Kapoor H, Arora N (2023). Resveratrol mitigates miR-212-3p mediated progression of diesel exhaust-induced pulmonary fibrosis by regulating SIRT1/FoxO3. Sci Total Environ.

[35] Wang Z, Yu T, Huang P (2016). Post-translational modifications of FOXO family proteins (review). Mol Med Rep.

[36] Li X, Liu H, Wang H (2014). Follistatin could promote the proliferation of duck primary myoblasts by activating PI3K/Akt/mTOR signalling. Biosci Rep.

[37] Puigserver P, Rhee J, Donovan J (2003). Insulin-regulated hepatic gluconeogenesis through FOXO1–PGC-1α interaction. Nature.

[38] Wu H, Olson EN (2002). Activation of the MEF2 transcription factor in skeletal muscles from myotonic mice. J Clin Invest.

[39] Chang S, Chen X, Huang Z (2018). Dietary sodium butyrate supplementation promotes oxidative fiber formation in mice. Anim Biotechnol.

[40] Kamei Y, Miura S, Suzuki M (2004). Skeletal muscle FOXO1 (FKHR) transgenic mice have less skeletal muscle mass, down-regulated type I (slow twitch/red muscle) fiber genes, and impaired glycemic control. J Biol Chem.

