# Supplementary material for: Resveratrol alleviated muscle fiber type transformation in oxidative stressed bovine myotubes through Akt/FoxO signaling pathway
Source: Anim Biosci. 2026 May 7;39(8):250976. doi: 10.5713/ab.250976 (PMC13441188; doi:10.5713/ab.250976)
Supplement: Supplementary file 1 [file ab-250976-Supplement-1.pdf]

**Supplement 1** Primers used for real-time quantitative PCR.

| Gene | Sequences (5′–3′) | Fragments size(bp) |
|---|---|---|
| *MyHC I* | F：ATCGCTGAATCCCAGGTCAA<br>R：ACCAAGATGTGGCACGGCTA | 92 |
| *MyHC IIa* | F：CACCCTGGAGCAGACAGAGA<br>R：TCCCTGGATTTGCGTGATG | 148 |
| *MyHC IIx* | F：AGGGCATCGCTGGAACAGAC<br>R：CAGAAGCTGCACACGCTCAC | 84 |
| *MyHC IIb* | F：TTTCCAGACCGTGTCTGCTC<br>R：GGGATGATGCAGCGTACAAAG | 96 |
| *PI3Kα* | F：CGGTGAGACTGTGTGGGACTTATC<br>R：CTGATGGAGTGTGTGGCTGTT | 115 |
| *PI3Kβ* | F：TTGGTGAGGATTCAGTTGGAGTG<br>R：AAGCAGCCGTAAGGTAGCATC | 142 |
| *Akt1* | F：CTCTCTGGGCTACTCAAGAAGGA<br>R：CACACGATGCTGGCGAAGAAT | 101 |
| *Akt2* | F：ATACCTTCGTCATTCGCTGTCTG<br>R：ATCCACTCTTCCCTCTCGTCTG | 88 |
| *FoxO1* | F：CTCCCACAGCAATGACGACT<br>R：CGTCATGATGGGAGAGAGCC | 100 |
| *FoxO3* | F：GCAGACCATCCAAGAGAACAAG<br>R：GCTGAGTGAGTCCGATGTGAG | 97 |

| | | |
|---|---|---|
| *PGC-1α* | F：GGAGCAATAAAGCGAAGAGCA<br>R：TGTGGGTTTGGTGTGAGGAG | 113 |
| *MEF2C* | F：AACCAGACCTCCGAGTTCTTATTC<br>R：GTAGCCAATGACTGAGCAGACT | 124 |
| *NRF-1* | F：CTGATGGCACTGTCTCGCTTAT<br>R：TGACTGTGGTTGGCAATTCTGA | 87 |
| *TFAM* | F：TGGGAAAGTCAGGAGCGGATC<br>R：TGGGCTTCTTTGGATAACCACTCA | 120 |
| *ATP6* | F：CACCCACTCCACTAATCCCAATAC<br>R：GCAAGTGTAGCTCCTCCGATT | 142 |
| *GAPDH* | F：GATGGTGAAGGTCGGAGTGAAC<br>R：GTCATTGATGGCGACGATGT | 100 |

MyHC, myosin heavy chain; PI3K, phosphatidylinositol3-kinase; Akt, protein kinase B; FoxO, forkhead box protein O; PGC-1α, peroxisome proliferator-activated receptor gamma coactivator-1α; MEF2C, myocyte Enhancer Factor 2C; NRF-1, nuclear respiratory factor 1; TFAM, mitochondrial transcription factor A; ATP6, adenosine triphosphate 6; GAPDH, glyceraldehyde-3-phosphate.
